# Infant development in family context: call for a genetically informed approach

**DOI:** 10.3389/fgene.2012.00167

**Published:** 2012-09-03

**Authors:** Stephanie H. Parade, John McGeary, Ronald Seifer, Valerie Knopik

**Affiliations:** ^1^Bradley/Hasbro Children's Research Center and the Department of Psychiatry and Human Behavior, Warren Alpert Medical School, Brown University, ProvidenceRI, USA; ^2^Providence Veterans Affairs Medical Center and Center for Alcohol and Addiction Studies, Brown University, ProvidenceRI, USA; ^3^Division of Behavioral Genetics, Department of Psychiatry, Rhode Island Hospital, ProvidenceRI, USA

**Keywords:** family functioning, infancy, temperament, gene-environment interactions, development

## Abstract

We call for a genetically informed approach in the examination of infant social and emotional development in family context. We recommend that scholars conceptualize family functioning as occurring on three unique levels: the parent-child dyad, the inter-parental dyad, and whole family functioning. Although advances in the area of understanding genetic variation in infants as a potential moderator of the influence of parent-child dyadic functioning have been made over the past decade, it is time to widen this inquiry to consider genetic variation in infants as a potential moderator of the influence of inter-parental dyadic and whole family functioning as well. A critical review of the literature also calls for additional examination of genetic variation in infants as a moderator of positive contextual influences, the integration of unique temperament variables with studies of infant genotype, consideration of the role of the gene-environment correlation, and epigenetic effects. Furthermore, we call for the application of genetically-informed research methods to these questions. Expanding knowledge in this area has the potential to refine treatment and prevention efforts aimed at promoting infant social and emotional development.

## Infant development in family context: call for a genetically informed approach

The family is the most proximal context for infant development. Infants reared in dysfunctional family environments are at risk for deficits in their social and emotional health; however, not all infants who are exposed to family dysfunction experience negative outcomes. Theories of gene-environment interactions, diathesis-stress, and differential susceptibility propose that some individuals are more likely to be influenced and altered by their environments than are others due to their individual characteristics including their temperamental reactivity and genetic makeup (Monroe and Simons, 1991; Boyce and Ellis, 2005; Belsky and Pluess, 2009). Infant temperamental reactivity, defined as the intensity and duration of an infant's reaction to novel, frustrating, or challenging situations (Rothbart and Derryberry, 1981), moderates the influence of family functioning on infants' social and emotional development. We call for greater attention to genetic variation in infants as a potential moderator of the influence of family functioning as well.

Infancy is a critical period in which to examine links between family functioning and infant social and emotional health. Experiences within the family in infancy are postulated to lay the foundation for experiences in subsequent stages of development (Sameroff and Chandler, 1975), and to contribute to trajectories of development which may be characterized by adaptive development or dysfunction (Crockenberg and Leerkes, 2000). However, not all infants who are exposed to dysfunctional family contexts experience later negative outcomes. Understanding the role of gene-environment interactions in infancy has the potential to inform understanding of why some individuals fare better than others despite experiences of stress within the family in the earliest years of life.

Since Crockenberg's (1981) landmark study demonstrating that maternal social support exerts a stronger effect on infant attachment security among highly irritable than less irritable infants, interest in infant characteristics which moderate family influence on infant developmental outcomes has risen steadily. Infant temperamental reactivity has long been recognized as a moderator of the influence of family functioning on infant social and emotional development, and more recent work demonstrates genetic variation in infants as a moderator as well. This is not surprising given that temperament is generally regarded as biologically based with individual differences in components of temperament being rooted in genotype (Fox et al., 2008). Therefore, rather than being distinct characteristics, to the extent that reliable relationships between the two are seen, temperament and genotype may be regarded as interdependent. Moderation effects between individual characteristics of the infant (such as temperament and genotype) and the family context have historically been interpreted in light of transactional (Sameroff, 1975) or diathesis-stress (Monroe and Simons, 1991) perspectives which emphasize individual characteristics as risk and protective factors which enhance or buffer effects of environmental risk. More recently, emphasis has been placed on differential susceptibility (Belsky and Pluess, 2009) and biological sensitivity to context (Boyce and Ellis, 2005) perspectives which conceptualize individual characteristics as markers of susceptibility to environmental influences. The later perspectives suggest that trajectories of development are not only more likely be modified by negative contextual influences for those highly susceptible individuals, but are more likely to modified by positive contextual influences for highly susceptible individuals as well. Although evidence of differential or biological sensitivity to context is emerging, it remains to be seen if highly susceptible individuals are truly more likely to be influenced by positive family contexts than less susceptible individuals. However, accumulating evidence does support transactional and diathesis-stress perspectives that infant temperamental reactivity moderates the influence of dysfunctional family functioning on infants' social and emotional adjustment as reviewed below. We call for greater attention to genetic variation in infants as a potential moderator as well.

Family functioning may be conceptualized as occurring at three interdependent yet unique levels: the parent-child dyad, the inter-parental dyad, and whole family functioning (Hayden et al., 1998). Each of these levels of family functioning represent various subsystems within the family which have unique patterns of interaction, rules, and boundaries (Minuchin, 1985; Cox and Paley, 1997), and all three levels of family functioning exert a unique influence on infant social and emotional development. At the level of the *parent-child dyad*, infants whose mothers respond less sensitively to their distress signals develop fewer adaptive emotion regulation strategies (Crockenberg and Leerkes, 2004; Jahromi and Stifter, 2007) and more behavior problems and less social competence in toddlerhood (Leerkes et al., 2009). Further, a secure attachment to parents in infancy is a precursor of a range of adaptive outcomes in later infancy, toddlerhood, and childhood (see Thompson, 2008 for a review). At the level of the *inter-parental dyad*, infants who are exposed to more maladaptive conflict strategies between parenting partners are more likely to utilize maladaptive and less likely to utilize adaptive strategies to regulate their own negative emotions (Porter et al., 2003; Crockenberg et al., 2007; Parade and Leerkes, 2011). Infants who are exposed to more inter-parental conflict also experience more atypical patterns of vagal regulation when they are interacting with their mothers compared with infants who are exposed to less inter-parental conflict (Moore, 2010). Furthermore, paternal marital satisfaction is positively associated with infant visual referencing to both mothers and fathers during times of ambiguity (Dickstein and Parke, 1988), underscoring the potentially positive and negative effects of inter-parental dyadic functioning for infant social and emotional development. At the level of the *whole family*, whole family functioning is positively associated with a secure infant attachment style (Dickstein et al., 2009). Despite the importance of each unique level of family functioning for infant social and emotional health, not all infants who are exposed to family dysfunction experience negative outcomes. Associations between each aspect of family functioning and infant social and emotional outcomes tend to be small to moderate in strength suggesting that some infants are more susceptible to dysfunctional family contexts than are others due to individual characteristics, including temperamental reactivity and genotype.

## High temperamental reactivity as a moderator of family functioning

Infant temperamental reactivity moderates links between all three levels of family functioning (inter-parental, parent-child, and whole family) and infant outcomes. At the level of the *parent-child dyad*, evidence supports diathesis-stress (Monroe and Simons, 1991) as well as differential susceptibility (Belsky and Pluess, 2009) and biological sensitivity to context (Boyce and Ellis, 2005) perspectives. Maternal responsiveness and sensitivity to infant distress is linked with less affect dysregulation and more receptive cooperation among infants who are highly temperamentally reactive (Kochanska et al., 2005; Leerkes et al., 2009), and affect synchrony in mother-infant interactions is more strongly associated with infant self control at age 2 among infants with more difficult temperaments than infants with less difficult temperaments (Feldman et al., 1999). The moderating effect of infant temperamental reactivity extends into later childhood, links between parenting in infancy and children's academic competence, social skills, and relations with peers and teachers in the first grade are stronger among infants with more difficult temperaments than infants with less difficult temperaments (Stright et al., 2008), and children with more difficult temperaments are more susceptible to both positive and negative maternal discipline than children with less difficult temperaments (Van Zeijl et al., 2007).

With regard to the level of the *inter-parental dyad*, inter-parental aggression is negatively associated with adaptive infant emotion regulation only among infants who are highly temperamentally reactive to fear (Parade and Leerkes, 2011), and associations between inter-parental conflict and behavior problems in later toddlerhood are strongest among children rated high in negative emotionality, a correlate of temperamental reactivity, at 4 months (Pauli-Pott and Beckmann, 2007). There is less evidence to support differential susceptibility (Belsky and Pluess, 2009) and biological sensitivity to context (Boyce and Ellis, 2005) perspectives with regard to infant susceptibility to positive inter-parental dyadic functioning, highlighting a need for additional research in this area.

Finally, at the level of the *whole family*, conflict within the family is associated with internalizing and externalizing behavior problems only among preschoolers with more difficult temperaments (Tschann et al., 1996). This moderation effect is characteristic of the link between family conflict and externalizing behavior problems in later childhood as well (Ramos et al., 2005). Few studies have examined infant temperament as a moderator of adaptive family contexts to provide support for the perspective that some infants are more susceptible to positive aspects of whole family functioning as well. Taken together this body of research provides support for infant temperamental reactivity, specifically high reactivity, as a factor associated with infant susceptibility to the influence of family functioning on social and emotional development.

## Genetic variation in infants as a moderator of family functioning

Despite support for infant temperamental reactivity as moderator of the influence of all three levels of family functioning on infant outcomes, less is known about the moderating role of genetic influences. Investigations in this area thus far have been limited to single locus association studies. This approach, despite its limitations, is a necessary starting point to establish the presence of candidate genes and provide a springboard for more sophisticated genetically-informed methodologies (e.g., the aggregation of individual polymorphisms to create a susceptibility score). Therefore, we review the current evidence for genes associated with heightened susceptibility to environmental influences and suggest future directions for this field of research. The first evidence of genetic influences on susceptibility to environmental influences identified specific candidate genes associated with behavioral indices of temperamental reactivity and negative emotionality (Belsky and Pluess, 2009; Caspi et al., 2010) including the serotonin-transporter gene *SLC6A4*, the D4 dopamine receptor gene *DRD4*, the brain-derived neurotrophic factor *BDNF* val66met polymorphism (Jiang et al., 2005), and the corticotrophin-releasing hormone (*CRH*) gene (Smoller et al., 2003, 2005). To date, the majority of research examining infant genetic susceptibility to family functioning has focused on *SLC6A4* and *DRD4* and we present this research briefly here.

The triallelic *5-HTTLPR* polymorphism is the most commonly investigated polymorphism of *SLC6A4* and is associated with differential uptake of serotonin in the synapse. Individuals with the low expressing alleles of *5-HTTLPR* (S and L_G_) have reduced uptake of serotonin as compared to individuals with the high expressing allele (L_A_) and such individuals have elevated risk for depression and anxiety (Lucki, 1998; Ressler and Nemeroff, 2000). Among adults, the low expressing alleles are also associated with a greater attentional bias to emotional stimuli (Beevers et al., 2007, 2009) and with less emotional resilience in the face of adversity (Stein et al., 2009). In infancy, the low expressing alleles are associated with heightened negative emotionality (Auerbach et al., 1999; Lakatos et al., 2003). This suggests that *5-HTTLPR* may moderate effects of family functioning on infant social and emotional development.

The exon 3 VNTR polymorphism in the D4 dopamine receptor gene *DRD4* is associated with receptor efficiency in binding dopamine. Individuals with the long alleles are less efficient in binding dopamine than individuals with the short alleles, and adults with the long alleles exhibit greater novelty seeking and impulsivity (Ebstein et al., 1996; Ebstein, 2006). In infancy, the long alleles are associated with more negative affect and greater activity level (Auerbach et al., 2001; Holmboe et al., 2011). Consequently, this *DRD4* polymorphism may moderate effects of family functioning on infant social and emotional development as well.

At the level of the *parent-child dyad*, both *SLC6A4* and *DRD4* variation moderates links between characteristics of the parent-child relationship and infant social and emotional development. Low parental responsiveness and sensitivity are risk factors for an insecure infant attachment style only among infants with the low expressing alleles of the *5-HTTLPR* polymorphism of *SLC6A4* (Barry et al., 2008), and an insecure infant-parent attachment style is a risk factor for maladaptive emotion regulation only among infants with the low expressing alleles of the *5-HTTLPR* polymorphism (Kochanska et al., 2009). In later childhood, high levels of maternal criticism contribute to children's attentional avoidance of anger, but only among children with the low expressing alleles of *5-HTTLPR* (Gibb et al., 2011). The *DRD4* exon 3 VNTR moderates links between aspects of the *parent-child dyad* and infant outcomes as well. Maternal sensitivity in infancy is negatively associated with the development of externalizing behavior problems only among infants with the long allele of this *DRD4* polymorphism (Bakermans-Kranenburg and Van Ijzendoorn, 2006), and an aggregate measure of parenting quality is associated with sensation seeking only among infants with the long allele (Sheese et al., 2007). Likewise, a family intervention designed to promote sensitive parenting exerts a positive influence on HPA axis functioning of infants with the long allele of the *DRD4* exon 3 VNTR (Bakermans-Kranenburg et al., 2008a,b), and children with the long allele appear to benefit most, with regard to declines in externalizing behavior problems, from increased maternal positive discipline strategies resulting from intervention as well (Bakermans-Kranenburg et al., 2008a,b). Finally, parent-child attachment security at age 7 is associated with children's donating behavior, indexed by the number of coins children donated to a charity in a standardized laboratory protocol, but only among children with the long allele (Bakermans-Kranenburg and Van Ijzendoorn, 2011). A secure attachment style is associated with greater donating behavior whereas an insecure attachment style is associated with less donating behavior. This collective body of research provides support for the perspective that not only are some individuals more susceptible to contextual stress (Monroe and Simons, 1991), but are more susceptible to the effects of positive environments as well (Boyce and Ellis, 2005; Belsky and Pluess, 2009).

To our knowledge, no previous studies have specifically examined infant susceptibility to family functioning at the level of the *inter-parental dyad* or the level of *whole family functioning*, yet research investigating the role of maternal social support does support the potential moderating effect of genetic variation in infants. Maternal reports of social support are associated with behavioral inhibition at age 7 only among children with the low expressing alleles of *5-HTTLPR* (Fox et al., 2005). The measure of social support utilized in this previous research has demonstrated associations with measures of whole family and dyadic functioning which are moderate to large in magnitude (Weinert and Tilden, 1990), supporting the possibility that *5-HTTLPR* potentially moderates links between these aspects of family functioning and infant social and emotional development as well. It will be important for future research to consider genetic variation in infants as a potential moderator of each unique level of family functioning independently rather than combining levels of family functioning into a single variable to form a composite. It is possible that some infant genotypes more strongly moderate the influence of some levels of family functioning as opposed to others, yet this remains to be seen.

The dearth of previous research examining genetic variation in infants as a moderator of the influence of both the inter-parental dyad and whole family functioning represents a significant gap in knowledge of the impact of the family environment for infant social and emotional development. Understanding the influence of genetics in these links is important for the development and validation of prevention and intervention programs to promote more adaptive family environments in infancy by identifying conditions under which family dysfunction is a risk factor for deficits in infant social and emotional development. It is possible that intervention and prevention programs designed to improve whole family functioning and inter-parental dyadic functioning are particularly beneficial for infants whose genotype confers heightened susceptibility to environmental influences. Supporting this possibility, interventions to enhance parenting appear most beneficial for infants with the *DRD4* exon 3 VNTR alleles associated with heightened susceptibility to environmental influences as described above (Bakermans-Kranenburg et al., 2008a,b). Rather than using this knowledge to “select” individuals who will receive prevention and intervention programs, an understanding that some children may benefit more than others based upon their unique characteristics is an important consideration when evaluating program efficacy. Mean effect sizes for program efficacy may vary across groups of children who are more or less susceptible to environmental influence, and small effect sizes which are characteristic of the majority of prevention and intervention programs may have substantial outcomes for some children.

## Considerations and recommendations for future research

A critical review of the literature with regard to genetic variation in infants as a moderator of the influence of each unique level of family functioning highlights numerous opportunities for research in this area above and beyond conducting additional research in the domains of inter-parental dyadic and whole family functioning.

First, the work highlighted in the review is generally consistent with transactional (Sameroff, 1975) and diathesis-stress (Monroe and Simons, 1991) perspectives which emphasize individual characteristics as moderators of environmental risk. Less work has been conducted to determine if individual characteristics make some individuals more susceptible to positive family contexts consistent with differential susceptibility (Belsky and Pluess, 2009) and biological sensitivity to context (Boyce and Ellis, 2005) perspectives. This is especially true for the examination of infant susceptibility to positive inter-parental dyadic and whole family functioning, as the majority of work examining susceptibility to positive aspects of family functioning has been at the level of the parent-child dyad. This may be in part because studies of human development tend to focus on sequelae of risk rather than adaptive functioning. As advocated by others (e.g., Belsky et al., 2009) further examination of the outcomes of positive developmental contexts, and whether individual characteristics including temperament and genotype moderate those links, is important. Understanding if some individuals are more susceptible to positive outcomes than others may help in understanding why some infants benefit more than others from intervention.

Second, a critical review of the literature highlights not only evidence supporting infant temperamental reactivity and genotype as moderators of the influence of family functioning, but also non-replications of these interaction effects as well. As this is a common limitation of the candidate gene literature, it is recommended that interaction effects should be cautiously interpreted until they have been replicated across samples (Rutter, 2006). Indeed, in two large samples utilizing gold standard assessments of maternal sensitivity and infant attachment security, *SLC6A4* and *DRD4* polymorphisms did not consistently moderate links between maternal sensitivity and infant attachment security (Luijk et al., 2011). Importantly, this is a limitation of the larger developmental literature with regard to infant temperament as well. For example, although some studies have demonstrated evidence that infant temperament moderates effects of inter-parental dyadic functioning (e.g., Pauli-Pott and Beckmann, 2007; Parade and Leerkes, 2011), in others infant temperament has not emerged as a consistent moderator (e.g., Crockenberg et al., 2007). And, in a sample of older children inter-parental conflict was more strongly associated with internalizing and externalizing behavior problems among children who exhibited low fear and sadness (El-Sheikh, 2005). Replication of moderation effects of temperament is therefore necessary as well. This issue is further extended by the “file drawer” problem of unpublished non-replications of these interaction effects. This suggests that the utilization of replication samples in the examination of genetic variation and temperament as moderators of the influence of family functioning would be optimal.

Third, the examination of genetic variation in infants as a moderator of the influence of family functioning on infant social and emotional development should concurrently examine infant temperament as a moderator as well to determine if temperament and genotype exert similar moderating effects. Rather than focusing on the broad dimension of infant temperamental reactivity or negative emotionality as is characteristic by much of the literature reviewed here, examining unique temperament variables including frustration and fearfulness would be advantageous given that these variables are associated with unique neurobiological systems (Rothbart et al., 1994; Rothbart and Bates, 2006). Understanding if unique temperamental characteristics, as opposed to broad dimensions of temperament, are particularly salient moderators will guide the selection of future candidate genes to test as moderators of the influence of family functioning.

Fourth, gene-environment interactions highlighted in this review reflect infant susceptibility to environmental influences; however, it is important to acknowledge that when examining infant susceptibility to family functioning that these associations are likely complicated by gene-environment correlation (rGE), which reflects differences in environmental exposure based upon genetic makeup (Jaffee and Price, 2007). That is, family functioning is not purely environmental, but rather reflects genetic influences from both the parent and child. rGE may be considered from the perspective of the parent or child, with either the parent or child's genes serving as the unit of measurement (Horwitz and Neiderhiser, 2011). In the current review, we focus on the child's genes as the unit of analysis. Children have long been recognized to influence their family environment (Sameroff, 1975; Belsky, 1984). Three types of rGE have been identified including passive rGE, evocative rGE, and selective rGE (Jaffee and Price, 2007; Horwitz and Neiderhiser, 2011). Passive rGE is the result of both shared genes and environment between infants and their parents. For example, infants who are highly temperamentally reactive may acquire that trait from their parents, and may be exposed to dysfunctional family environments (at all three levels: whole family functioning and inter-parental and parent-child dyadic functioning) due to their parents' tendency to be highly temperamentally reactive. Evocative rGE is the result of an individual's genetic makeup evoking a response from the environment. In this case, infants who are highly temperamentally reactive (genetically influenced) may elicit more negative parental behavior, a distressed inter-parental relationship, and more dysfunctional whole family functioning than infants who are less temperamentally reactive. Active rGE is the result of an individual selecting a particular environment due to their genetic makeup. For example, infants who are highly temperamentally reactive may indirectly “select” childcare environments with particular characteristics if their parents are sensitive to their needs. More specifically, parents who are aware that their infants are highly temperamentally reactive may place their infants in childcare settings with caregivers who are more responsive to infant reactivity. Family-based studies, including those that have utilized Children of Twins (Rutter et al., 2001) and Extended Children of Twins (Narusyte et al., 2008) designs, have detected rGE with indicators of family functioning (for a review see Horwitz and Neiderhiser, 2011). Molecular rGE are emerging in the literature (for a review see Jaffee and Price, 2007). rGE may account for many of the gene-environment interactions highlighted in this review. Family-based designs that account for both parental and offspring influences (both genetic and environmental), and genetically-informed adoption designs which account for passive rGE (Haugaard and Hazan, 2003; Leve et al., 2007), would be an important extension of the existing research on genetic variation in infants as a moderator of the influence of family functioning.

Fifth, it is important to acknowledge that epigenetic mechanisms may complicate efforts to investigate genetic moderation of effects of family functioning as well (Fagiolini et al., 2009; Murgatroyd et al., 2009). Epigenetic modification may not only alter gene expression in a fashion that would complicate moderation of the influence of family functioning, but epigenetic modification can also be differentially manifested in individuals depending upon how strongly they are impacted by environmental influences. DNA methylation is perhaps the most commonly studied epigenetic mechanism and increases in methylation are typically associated with reductions in gene expression (Egger et al., 2004; Reik, 2007; Uddin et al., 2011). Consequently, DNA methylation may alter the expression of genes which moderate effects of family functioning. Supporting this possibility, increased methylation of the serotonin transporter *5-HTT* gene exacerbates links between maternal deprivation in infancy and behavioral stress reactivity among non-human primates (Kinnally et al., 2010). An additional complexity of the role of epigenetics in these links is emerging evidence that stressors in early childhood including inter-parental violence, parental depression, and socio-economic stress, are predictive of DNA methylation in adolescence (Essex et al., 2011; Radtke et al., 2011). This suggests that not only may epigenetic mechanisms contribute to an infant's relative level of susceptibility to family functioning, but that family functioning may also contribute to the epigenetic mechanisms themselves. Although not the focus of the current review, an awareness of the relevance of epigenetic mechanisms in the examination of infant susceptibility to family functioning is critical.

Finally, just as numerous opportunities exist to expand understanding of the role of genetic variation in infants in the influence of three unique levels of family functioning, so too do new frontiers exist in the application of genetically-informed research methods to these questions including: quantitative genetic modeling, genomic scale interrogation of genetic influences, systems-based aggregate genetic approaches, and family-based designs. Quantitative genetic approaches using data from infancy may highlight the respective contributions of genetic and environmental influences that are common or unique to these distinct phenotypes (i.e., reactivity to the three levels of familial functioning). Additionally, given emerging evidence consistent with the existence of susceptibility genes within the so-called “usual suspects” in psychiatric genetics (e.g., 5-HTTLPR, DRD4 exon 3 VNTR, etc.) an agnostic genomic level approach may reveal additional markers of infant susceptibility to family functioning. Identification of individual markers (as reviewed above) further suggests that a systems-based approach to aggregating genetic susceptibility may have utility. While these approaches are currently in their infancy, the premise of developing cumulative indices may clarify mixed research findings of single variants (i.e., by partially addressing differences in genetic background). These approaches may also begin to account for a larger proportion of the variance in infant developmental outcomes without an inordinate loss of power. In sum, opportunities for refining understanding of genetic variation in infants as a moderator of all three levels of family functioning are coupled with extensive opportunities to leverage untapped genetically-informative approaches. Expanding knowledge in this area has the potential to refine treatment and prevention efforts aimed at promoting optimal infant social and emotional development.

The family has long been recognized as the most proximal context for infant social and emotional development, and individual infant characteristics are salient in this link. Infant temperamental reactivity moderates the influence of family functioning on infants' social and emotional adjustment, yet less is known about the moderating effect of genetic variation in infants, despite the fact that temperament and its underlying genetic variation are interrelated. We call for a genetically informed approach in the examination of family functioning as it pertains to social and emotional development in infancy. To achieve this goal, we recommend that scholars conceptualize family functioning as occurring on three unique levels: the parent-child dyad, the inter-parental dyad, and whole family functioning. Examination of these three unique levels within a single study would be advantageous as well to deepen understanding if they exert influence on infant social and emotional development in unique ways. Advances in the investigation of genetic variation in infants as a moderator of the influence of parent-child dyadic functioning have been made over the past decade; it is time to widen this inquiry to consider genetic variation in infants as a moderator of the influence of inter-parental dyadic and whole family functioning as well.

### Conflict of interest statement

The authors declare that the research was conducted in the absence of any commercial or financial relationships that could be construed as a potential conflict of interest.
